# Tunicamycin promotes metastasis through upregulating endoplasmic reticulum stress induced GRP78 expression in thyroid carcinoma

**DOI:** 10.1186/s13578-020-00478-0

**Published:** 2020-10-01

**Authors:** Guohong Zhao, Jianqin Kang, Guanghui Xu, Jing Wei, Xiaoguang Wang, Xiaorui Jing, Lan Zhang, Aili Yang, Kai Wang, Jue Wang, Li Wang, Junfeng Hou, Qingquan Liu, Kai Jiao, Bin Gao

**Affiliations:** 1 Department of Endocrinology, Tangdu Hospital, Fourth Military Medical University, Xi’an, 710038 Shanxi China; 2 Department of Pediatrics, Tangdu Hospital, Fourth Military Medical University, Xi’an, 710038 Shanxi China; 3 Division of Digestive Surgery, Xijing Hospital of Digestive Diseases, Fourth Military Medical University, Xi’an, Shaanxi China; 4 Department of Ultrasound Diagnosis, Tangdu Hospital, Fourth Military Medical University, Xi’an, 710038 Shanxi China

**Keywords:** Tunicamycin, Metastasis, ER stress, GRP78, Thyroid cancer

## Abstract

**Background:**

Thyroid cancer (TC) is the most common type of endocrine malignancy and its incidence is increasing over years. Conventional surgery, radiotherapy and chemotherapy are difficult to improve the significant effects of it due to aggression and metastasis of poorly differentiated thyroid cancer (PDTC) and anaplastic thyroid cancer (ATC), and these are regarded as the most malignant types of TC. Glucose-regulated protein (GRP78) is the key molecule of tumor growth, apoptosis and metastasis. However, the underlying mechanisms of GRP78 in TC still require discussion. This study aimed to explore the role of GRP78 and its potential mechanism in TC.

**Results:**

GRP78 expression was increased in TC tissues when compared with adjacent normal tissues. Besides, down-regulation of GRP78 significantly inhibited the metastatic and proliferative ability of ATC cells in in vitro studies. In addition, tunicamycin-induced ER stress up-regulated the expression of GRP78, PERK and XBP1 as well as reversed the metastatic ability of GRP78 in ATC cells. Bioinformatics and statistical analysis of gene ontology (GO) enrichment and Kyoto Encyclopedia of Genes and Genomes (KEGG) pathways for RNA-sequencing data with regard to si-GRP78 and si-control showed that GRP78 might regulate the ability of metastasis through extracellular matrix (ECM) remodeling in ATC cells, as well as the expression of ECM components such as COL1A1 and MMP13, which were highly relevant to ATC cells. The analysis of GEPIA database confirmed that high genomic amplification of MMP13 and COL1A1 in TC tissues showed correlation with TNM stage. Further western blotting analysis showed that MMP13 might be the target of GRP78 in ATC cells and ER stress could activate the expression of MMP13 that is suppressed by GRP78 depletion.

**Conclusions:**

GRP78 acts as an important regulator of metastasis under ER stress. In addition, the function of GRP78 might be mediated by ECM remodeling in ATC cells, implicating it as a therapeutic target in TC.

## Background

Thyroid cancer (TC) is the most common malignancy of the endocrine system, and its global incidence rate has been increased by 20% over the last 20 years. The increasing incidence of it is probably due to the differences in diagnostic practices. In addition, environmental exposure to iodine levels and individual risk factors such as obesity also contribute to this cause [1]. Based on the degree of differentiation, TC is categorized into three kinds, which include well-differentiated thyroid carcinomas such as papillary thyroid carcinoma (PTC) and follicular thyroid carcinoma (FTC), as well as poorly-differentiated thyroid carcinoma (PDTC) and anaplastic thyroid cancer (ATC). Of these, PDTC and ATC accounted for only 15% and less than 5% of all TC cases [2]. These types of thyroid carcinomas are responsible for more than half of all TC mortalities that occur due to early lymph node (LN) metastasis and invasion of neighboring organs [3]. Standard treatments such as surgery, radiotherapy and chemotherapy are not shown to be successful in treating patients with advanced PDTC and ATC. Therefore, there is an urgent need for us to better understand the molecular mechanisms that underlie the pathogenesis of TC. The development of TC involves complex multiple genetic alterations that lead to the activation of numerous oncogenic genes and several major signaling pathways such as p53 mutation and PI3K/Akt/mTOR signaling pathway [4–6]. Several evidences have suggested the potential roles of BRAF, RAS, MMP2, MMP9 and TWIST in regulating the metastatic process of TC [7–11]. In addition, endoplasmic reticulum (ER) stress is believed to contribute to several other steps along with metastasis and proliferation process of various types of cancers. For example, ER proteins such as XBP1, PERK, ATF6 and ATF4 that are involved in ER stress have been reported to participate in tumor growth and metastasis [12–15]. However, the underlying mechanisms responsible for metastasis and proliferation of TC are still poorly understood. In general, new strategies to identify novel potential therapeutic targets in patients with TC should be explored.

Recent molecular and pathological studies have reported that glucose-regulated protein (GRP78) is involved in tumor development and progression [16–22]. GRP78, also known as HSPA5, is reported to be involved in ER stress and overexpression of many types of human cancers, such as hepatocellular carcinoma, esophageal cancer, gastric cancer and prostate cancer. Besides, high expression of GRP78 showed strong association with medullary thyroid carcinoma [16, 17]. Several studies have also shown that the expression of GRP78 is related to invasion and metastasis of different types of human cancers. Overexpression of GRP78 is related to increased lymph node (LN) metastasis and poor prognosis in patients with gastric cancer [18], while knockdown of it decreased the invasion and extracellular matrix (ECM) degradation in hepatocellular carcinoma cells [19]. These findings demonstrate that GRP78 might take part in tumor metastasis. However, the role of GRP78 in TC is not completely elucidated.

Hence, in the present study, RNA-seq and GO enrichment and KEGG pathways analyses were performed to explore the metastatic-related genes and pathways in ARO and FRO cells. Among these genes, the ECM components such as COL1A1 and MMP13 showed correlation with GRP78. As COL1A1 and MMP13 have been reported to be correlated to the metastasis of several types of cancers [23–25], GEPIA database was used to confirm the high expression of MMP13 and COL1A1 in TC tissues, showing correlated with TNM stage. Moreover, western blotting results showed that MMP13 might be a target of GRP78 in TC cells. These results indicate that GRP78 is a new agent that has the potential to reverse the metastasis under ER stress in TC cells. Of course, the potential function and mechanism of it in TC still remains to be elucidated in a more detailed manner.

## Results

### High expression levels of GRP78 in thyroid carcinoma

**Fig. 1:**
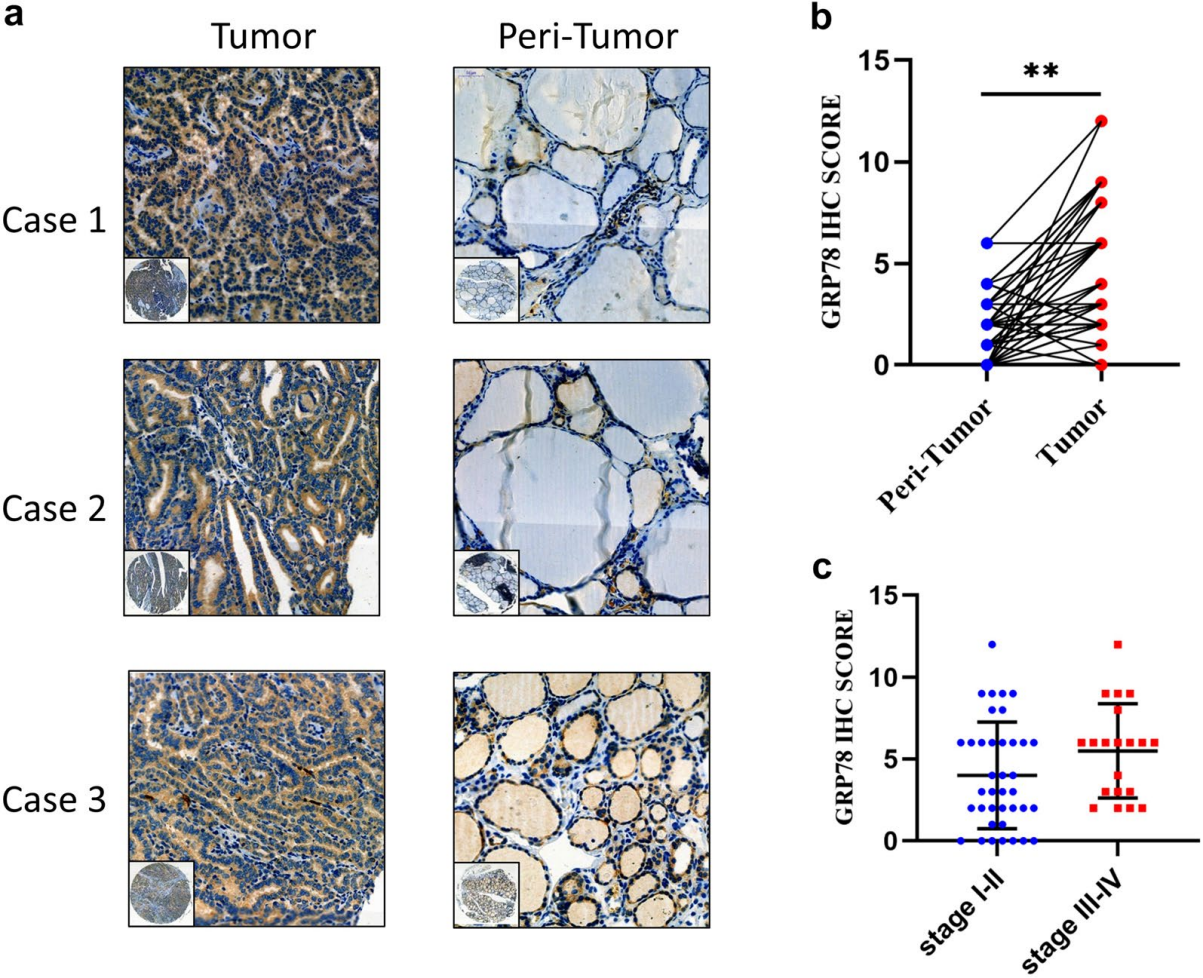
GRP78 expression is increased in thyroid carcinoma. **a** IHC was performed to compare the expression of GRP78 in TC tissues with peri-tumor tissues. **b** The scores of IHC showed increased levels of GRP78 in normal tissues when compared with peri-tumor tissues. ∗∗p < 0.01. **c** The scores of IHC showed the levels of GRP78 in stage I-II as compared with stage III-IV

The protein expression levels of GRP78 in thyroid carcinoma and peri-carcinoma tissues were examined by immunohistochemistry (IHC) assay. As shown in Fig. 1a, the immunoreactivity of GRP78 was observed in both cytoplasm and membranes of the tissues. The positive staining score of GRP78 in carcinoma tissues was significantly higher than that of the peri-carcinoma tissues (Fig. 1b). However, the expression of GRP78 showed no significant correlation in different stage (Fig. 1c). Due to aggressiveness and high mortality of ATC, two undifferentiated human thyroid carcinoma cell lines ARO and AFO were selected for the following test. Taken together, these findings suggest that the expression of GRP78 was higher in thyroid carcinoma.

### Down-regulation of GRP78 partly inhibits the migration and proliferation of thyroid cancer cells

**Fig. 2:**
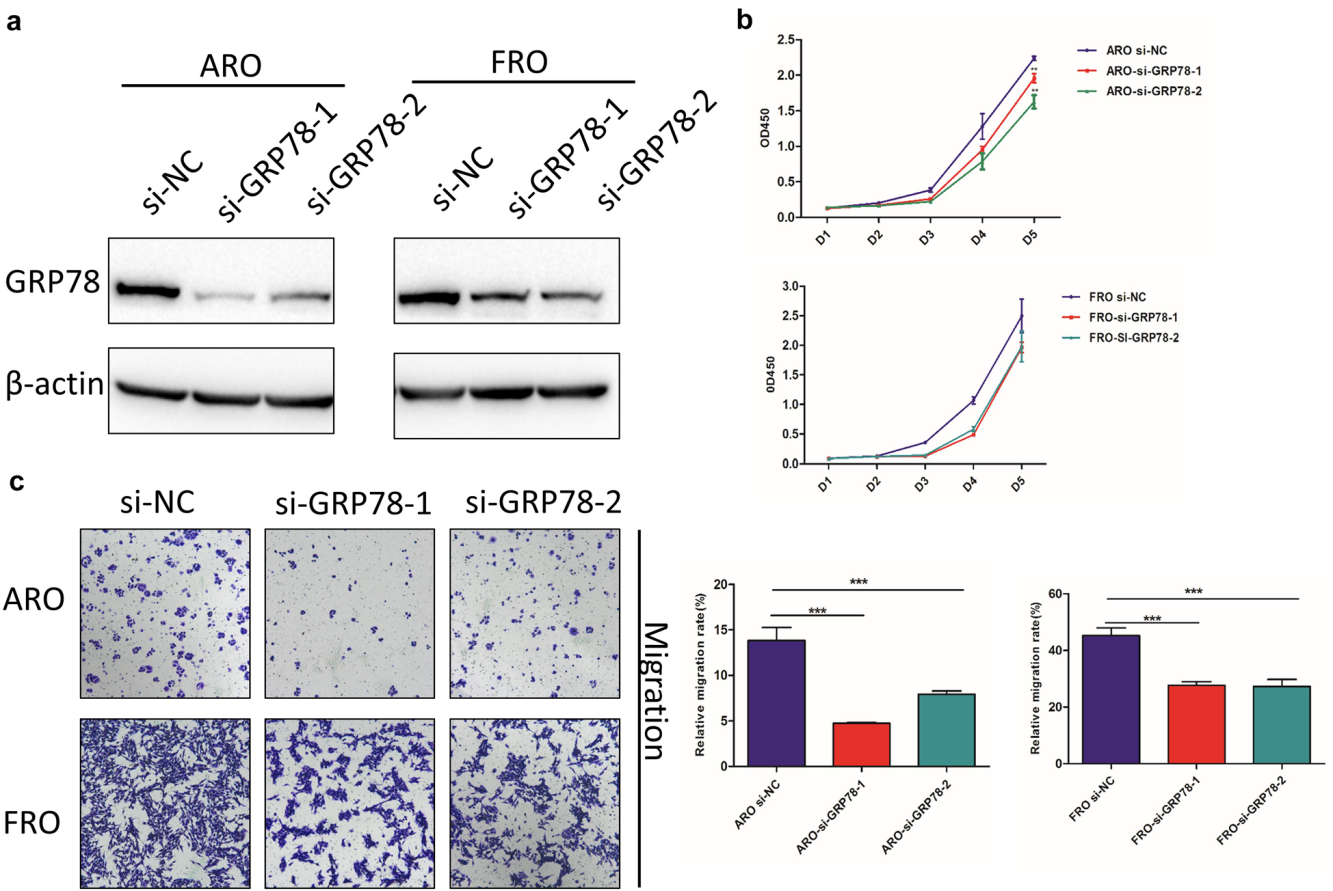
Knockdown of GRP78 suppresses proliferation and metastasis in ARO and FRO cells. **a** Western blotting analysis of GRP78 protein expression in ARO and FRO cells transfected with si-NC, si-GRP78-1 and si-GRP78-2. **b** CCK-8 proliferation assays were performed to determine the viability of siGRP78-transfected ARO and FRO cells. ∗∗p < 0.01. **c** Transwell assays were performed to assess the migration of siGRP78-transfected ARO and FRO cells. Bar graphs represent the average migration rate of ARO and AFO cells. ∗∗∗p < 0.001

To elucidate whether GRP78 was involved in TC cells, in vitro assays were performed to assess whether it plays a role in enhancing the proliferation and migration of TC cells, and so GRP78 siRNA was transfected into ARO and FRO cells. The results of western blotting analysis have confirmed the downregulation of GRP78 (Fig. 2a). The results of transwell assays showed that the migratory ability of GRP78 siRNA-transfected cells was significantly reduced when compared with control cells (Fig. 2c). In addition, CCK-8 assay was used to investigate the effects of GRP78 on proliferation inhibition, and the results also confirmed that proliferation of GRP78 siRNA-transfected cells was significantly inhibited when compared with control ones in ARO cells (Fig. 2b). Taken together, these results indicated that down-regulation of GRP78 can partly inhibit the migration and proliferation of ARO and FRO cells in vitro.

### Suppression of GRP78 expression partially decreases ER stress agonist-induced metastasis in TC cells

**Fig. 3:**
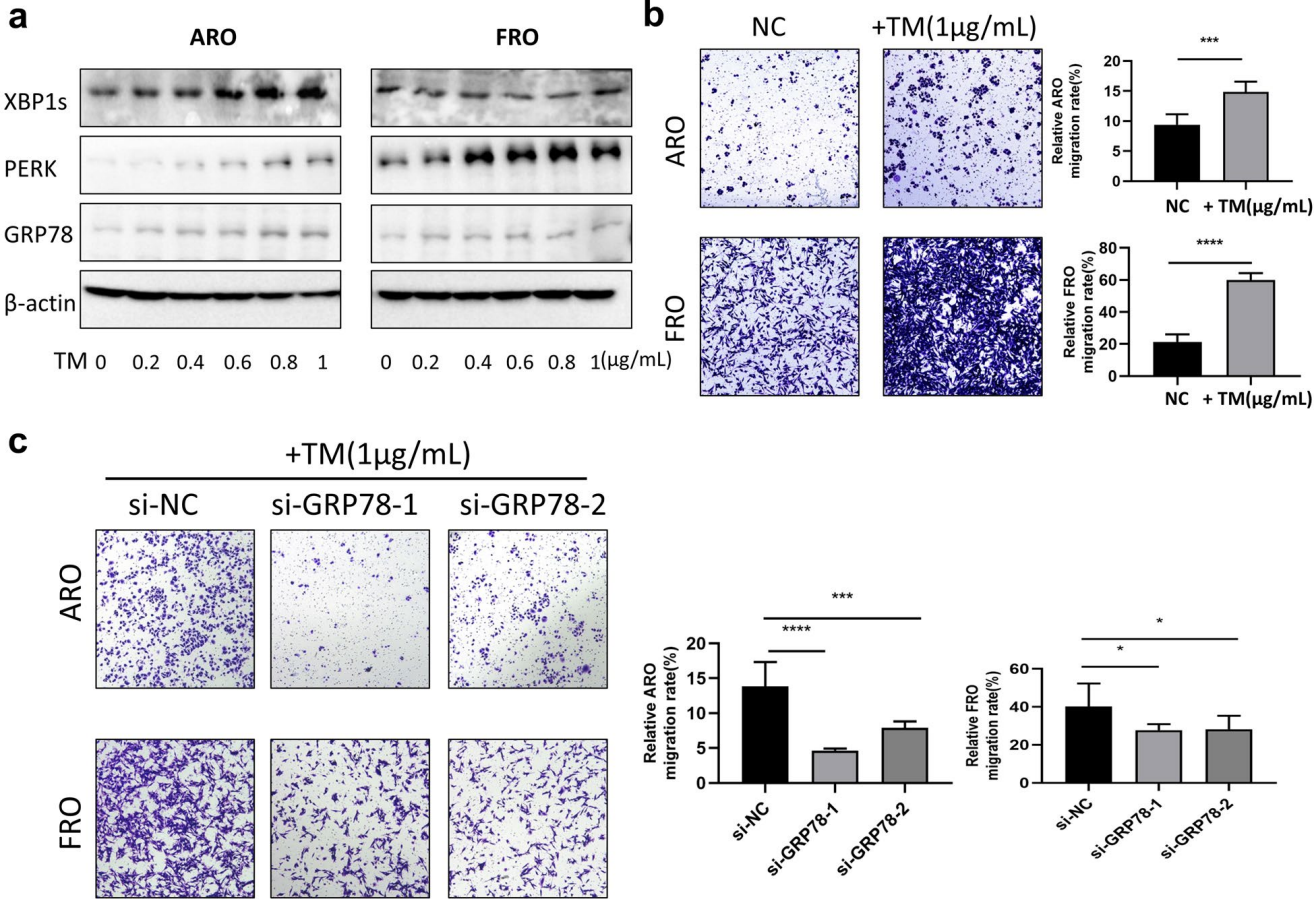
Suppression of GRP78 expression partially decreases the ER stress agonist-induced metastasis in thyroid cancer cells. **a** Western blotting analysis of XBP1s, PERK and GRP78 protein expression in ARO and FRO cells when treated with TM (0–1 μg/L). **b** Transwell assays were performed to assess the migration of ARO and FRO cells when treated with TM(1 μg/L). Bar graphs represent the average migration rate of ARO and AFO cells, ∗∗∗p < 0.001, ∗∗∗∗p < 0.0001. **c** Transwell assays were performed to assess the migration of siGRP78-transfected ARO and FRO cells when treated with TM(1 μg/L). Bar graphs represent the average migration rate of ARO and AFO cells, ∗p < 0.05, ∗∗∗p < 0.001, ∗∗∗∗p < 0.0001

The levels of expression of GRP78 showed correlation with metastasis in both ARO and FRO cells. This is because ER stress contributes to the metastasis of cancer cells. To understand the underlying molecular mechanism on GRP78-mediated suppression of metastasis in TC, in vitro assays were performed to determine whether GRP78 protein expression levels and the ability of metastasis are upregulated under ER stress. Both ARO and FRO cells were treated with different doses of TM (0–1 μg/mL). The results showed that TM (1 μg/mL) triggered ER stress and elevated the expression of GRP78, PERK and XBP1 in both cells by western blotting analysis (Fig. 3a), as well as TM-induced ER stress enhanced the ability of metastasis by transwell assays in both the cells (Fig. 3b). In addition, the metastatic ability that was found to be enhanced by TM-induced ER stress can be suppressed by the down-regulating GRP78 (Fig. 3c). These results suggest that ER stress induced upregulation of GRP78 was involved in enhancing the migration of TC cells.

### Depletion of GRP78 alters the expression of ECM related molecules and ECM remodeling pathway

**Fig. 4:**
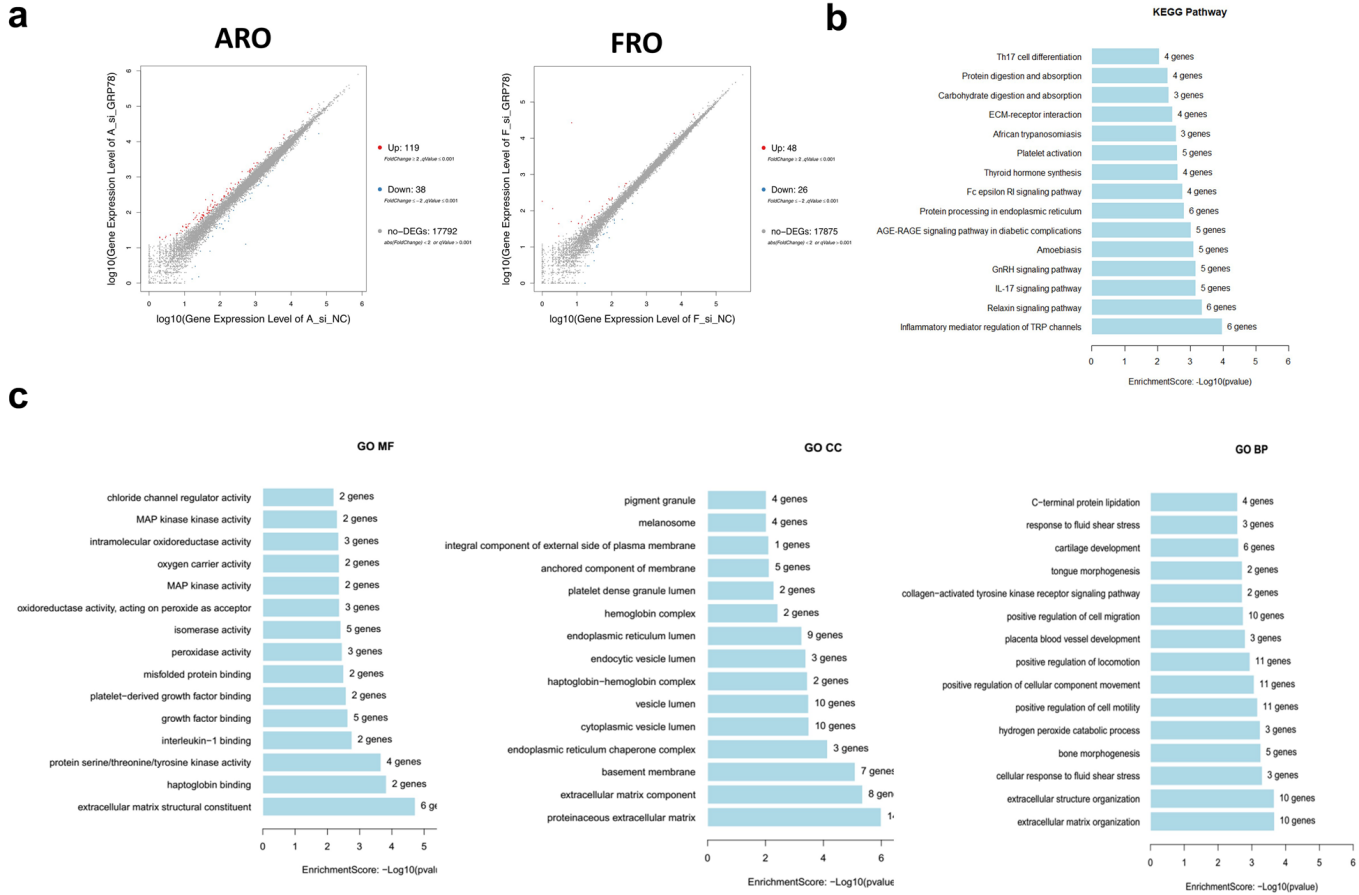
Depletion of GRP78 altered the expression of ECM related molecules and ECM remodeling pathway. **a** Volcano plot of differentially expressed mRNAs between normal si-NC and si-GRP78 of ARO and FRO cells. **b** Kyoto Encyclopedia of Genes and Genomes (KEGG) pathways analysis of DEGs of ARO and FRO cells. **c** GO enrichment terms and KEGG pathway analysis of DEGs of ARO and FRO cells. The statistically significant enriched GO terms in Biological Process, Cellular Component, and Molecular Function. The FDR corrected p values are displayed on a − log10 scale

The results described above have demonstrated that GRP78 could regulate metastasis by increasing the levels of ER stress in ATC cells. To further elucidate the underlying mechanisms with regard to the role of GRP78 in ATC cells, GRP78 and si-GRP78 were collected for performing RNA-sequencing in ARO and FRO cells. The results showed that 119 genes were upregulated and 38 genes were downregulated in ARO cells (Fig. 4a), while 48 genes were upregulated and 26 genes were downregulated in ARO cells (Fig. 4b). Furthermore, GO and the Kyoto Encyclopedia of Genes and Genomes (KEGG) enrichment analyses were performed to explore the functional roles of DEGs in ATC cells. GO analysis was performed and revealed that the location terms that were significantly over-represented in this set were the proteinaceous ECM, the ECM component and the basement membrane. In the GO analysis of molecular function and biological processes, one clear conclusion from RNA-seq analysis was drawn, in which GRP78 was found to be mainly related to the regulation of metastasis by ECM remodeling pathway in TC cells. Next, KEGG pathway analysis showed enrichment in a series of pathways, such as GnRH signaling pathway, ECM-receptor interaction and protein processing in the ER, and all of these are regarded critical in the process of tumor metastasis. In ECM-receptor interaction pathways, ECM components such as COL4A1, COL1A1 and MMP13 were shown to be highly relevant to TC.

### MMP13 is increased in thyroid carcinoma and might act as the target of GRP78

**Fig. 5:**
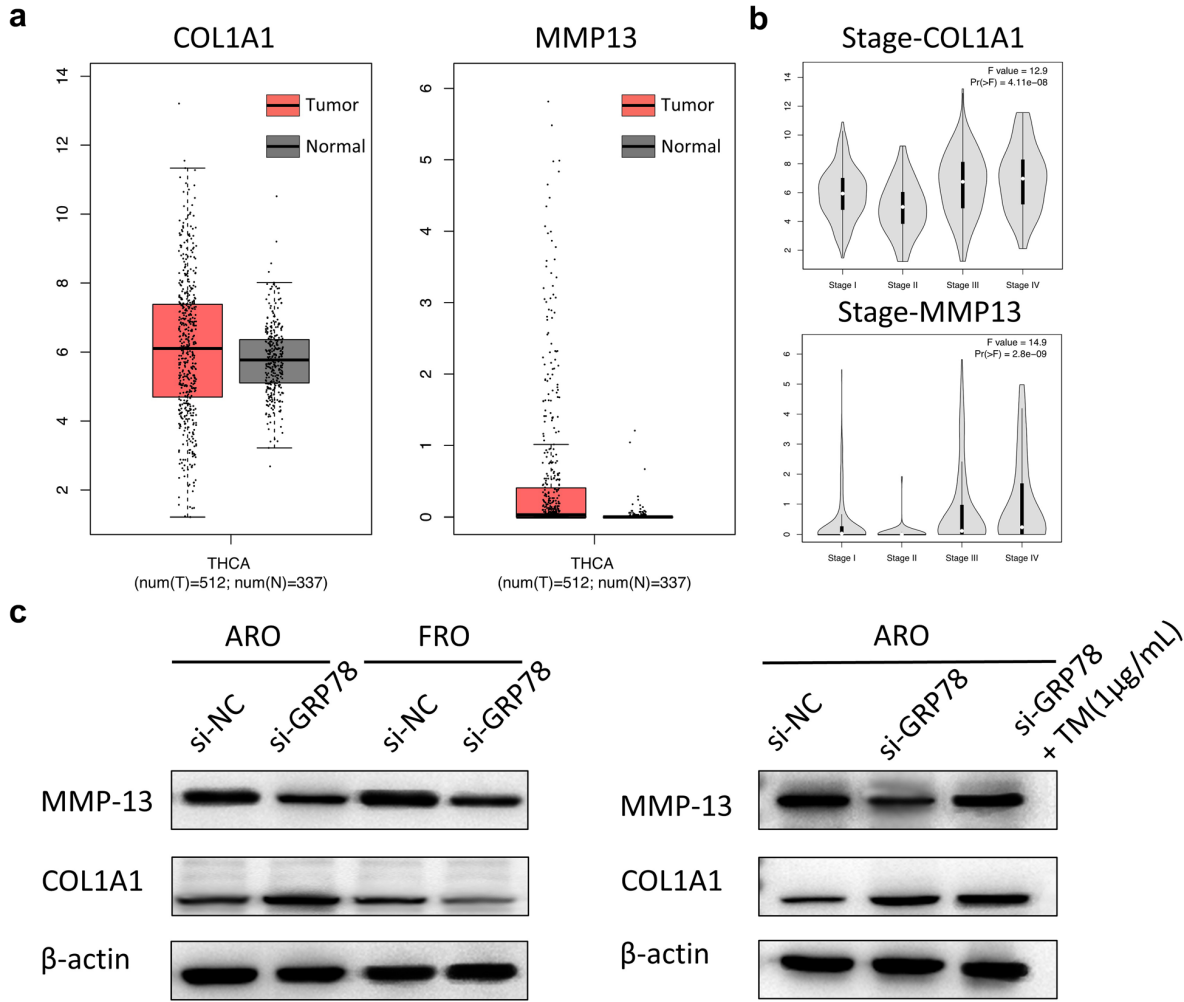
Analysis of the expression ofMMP13 and COL1A1 in ATC cells and MMP13 might be the target of GRP78. **a** Data from GEPIA database showed the mRNA expression of COL1A1 andMMP13 in normal tissues and TC tissues. Red boxes represent tumor tissues, and grey boxes represent normal tissues based on individual cancer stages. **b** Expression of COL1A1 andMMP13 in different stages of thyroid carcinoma. **c** Western blotting analysis of MMP13 and COL1A1 in GRP78 knockdown cells. **d** Western blotting analysis of MMP13 and COL1A1 in GRP78 knockdown cells when treated with TM (1 μg/L)

Based on the results of sequencing and bioinformatics analysis, MMP13 and COL1A1 were reported to contribute to the occurrence and development of tumor, especially the metastasis of tumor. To further verify the role of MMP13 and COL1A1 in TC, the data containing the differences in the expression of MMP13 and COL1A1 in 512 tumor tissues and 337 corresponding normal tissues were generated by using the Gene Expression Profiling Interactive Analysis (GEPIA) database. The results revealed high genomic amplification of MMP13 and COL1A1 in TC tissues (Fig. 5a). Moreover, genomic amplification of MMP13 and COL1A1 showed association with individual cancer stage (Fig. 5b). To further verify these results, western blotting was performed to determine whether the protein expression of MMP13 and COL1A1 showed similar variation. The results showed that depletion of GRP78 resulted in significant reduction of MMP13 expression in both the cells (Fig. 5c). In addition, ER stress could reverse this variation caused by down-regulating GRP78 in ARO cells (Fig. 5d). However, there was no significant trend on COL1A1, which further verified that MMP13 might be the target of GRP78. Of course, this needs to be further confirmed by experiments.

## Discussion

TC is the most commonly diagnosed endocrine malignancy, and its incidence is increasing over the past few decades throughout the world. PTC accounted for 80% of TCs, and showed a better prognosis. However, patients with PDTC or ATC due to rapid progression and local or distant metastasis frequently led to worse prognosis [1–4]. In addition to the characteristics of rapid growth of tumor cells, metastasis has been one of the hallmarks in cancer, leading to tumor dissemination and aggressiveness [26]. During this process, cancer cells become polarized, cross the surrounding ECM and stromal cell layers, and travel to distant sites, eventually growing into new metastatic colonies through cardiovascular or lymphatic circulatory systems [27]. Metastasis has become the main reason of curative failures in varied cancers and in cancer-related mortality that involves many molecules and pathways. Our previous research has shown that GRP78 is overexpressed and correlated with invasion, metastasis and poor prognosis of esophageal squamous cell carcinoma (ESCC) [20]. Besides, the expression of GRP78 is shown to be significantly higher in multidrug resistant gastric cancer cells and knockdown of GRP78 significantly reversed the multidrug resistance in gastric cancer [21, 22]. All these studies confirmed that GRP78 is involved in the pathological process of numerous cancers. Immunohistochemistry results of 62 PTC patients in our study revealed that the expression of GRP78 was significantly higher when compared with peri-carcinoma tissues in patients with PTC. However, patients with positive GRP78 expression showed no significant correlation with degree and differentiation. Thus, to elucidate whether the roles of GRP78 is involved in TC cells, transwell and CCK8 assays were performed and the results indicated that down-regulation of GRP78 could significantly inhibit migration and proliferation of ATC cells in vitro. Thus, our study results suggested that GRP78 promotes growth and migration of TC cells.

ER is a significant component of endomembrane system that modifies dysfunctional proteins and prevents them from secretion in eukaryotic cells. All the factors that affect the function of ER causes ER stress, and several other adverse conditions, such as glucose deprivation, acidosis, and severe hypoxia, and trigger ER stress, leading to the accumulation of misfolded proteins and production of unfolded protein response in ER. Growing evidence suggests that ER stress is not only a physiological state but also actively contributes to the occurrence and development of tumor. Upon ER stress, GRP78, which is also referred to as BiP (immunoglobulin heavy-chain binding protein), serves as an ER stress signaling regulator and plays critical role in the oncogenic stress. PERK and XBP1 are the most important ER membrane proteins that are recently linked to tumor cell migration/invasion processes such as ECM and EMT [28–30]. For example, overexpression of XBP1 could mediate EMT in HCC cells and the invasion and metastasis of HCC [28]. Similarly, PERK also contributes to ECM reorganization in breast cancer and overexpression of ATF4, which is a component of PERK pathway, induces cell invasion and metastasis, stimulating MMP2 and MMP7 expression in ESCC [13]. These evidences suggest that UPR activation might be relevant for the development of tumor metastasis. Till date, there are several ER stress inhibitors such as ATF6 inhibitor that were reported to display a strong effect on tumor migration inhibition of large range of cancer types including brain, breast, liver, lung, pancreas and skin. As part of UPR, the GRP78 protein could regulate multiple signaling pathways that showed association with metastasis, drug resistance, and immune function. To characterize the effect of ER stress on TC, TM was used as an ER inducer and the results showed that TM not only up-regulates the expression of GRP78, PERK and XBP1, but also promotes the ability of cell metastasis in ARO and FRO cells. Besides, downregulation of GRP78 could reverse the metastatic ability by TM-induced ER stress in ATC cells. These findings suggest GRP78 as a major molecular chaperone in the ER, promoting metastasis by TM-induced ER stress in ARO and FRO cells. However, more specific mechanisms should be studied further.

ECM is a complex dynamic structure that is present in all tissues and continuously undergoes degradation and remodeling. In addition to the structural support for cells, ECM not only interacts with cells through cell surface ECM receptors or other ways to regulate cell functions, such as proliferation, migration and differentiation [31]. Abnormal ECM remodeling and degradation could influence cell fate and behavior, resulting in some severe pathological conditions, such as fibrosis and invasive cancer [32]. Matrix metalloproteinases (MMPs) as extracellular or membrane-bound enzymes play critical roles in ECM remodeling [33]. A better understanding of how ECM remodeling affects disease progression contributes to the development of new therapeutic strategies. In our study, GO and KEGG enrichment analyses pathways were applied to screen DEGs and pathways in ATC cells, and these results suggest that GRP78 might regulate the ability of metastasis through ECM remodeling pathways in TC cells. Among these genes, ECM components such as COL1A1 (alpha-1 type I collagen) and MMP13 are obviously shown to be related to ECM remodeling pathway. Previous investigations have indicated that COL1A1 and MMP13 are associated with cancer metastasis, and TM-induced apoptosis also showed close association with GRP78 and MMP13 in renal carcinoma cells [23–25, 34]. To investigate the effects of COL1A1 and MMP13 in TC, the data showed differential expression of MMP-13 and COL1A1 in 501 tumor tissues and 58 adjacent normal tissues generated by using GEPIA datasets (Gene Expression Profiling Interactive Analysis) [35]. These results revealed that high genomic amplification of MMP13 and COL1A1 in TC tissues and overexpression of MMP-13 and COL1A1 are related to advanced TNM staging. Besides, ER stress activates the expression of MMP-13, which was suppressed by depletion of GRP78. More accurate experiments are further warranted to verify these results.

## Conclusion

In summary, the results of this study revealed that the mechanism of GRP78 mediated ER stress-metastasis in ATC cells showed association with ECM remodeling pathway. Effective overexpression of GRP78 remarkably promoted ER stress-metastasis. Of note, GRP78 might also promote metastasis via regulating the expression of MMP-13. Better understanding of the mechanism allows us to more specifically target relevant factors that prevent tumor metastasis and improve novel therapeutic targets in patients with TC.

## Methods

### Cell culture and TC tissues

The undifferentiated ATC cell lines ARO and FRO were initially obtained from the Chinese Academy of Medical Science. All cell lines were maintained in RPMI 1640 medium (Gibco, USA) supplemented with 10% fetal bovine serum (FBS, ZETA, USA) at 37 °C in a humidified atmosphere containing 5% CO_2_. The cells in the logarithmic phase of growth were used throughout the experiment. The TC tissue microarrays were obtained from Shanghai Outdo Biotech Company (Shanghai, China). Each array contained TC tissues and adjacent TC tissues from a total of 62 cases.

### Immunohistochemical analysis (IHC) and pathology scores

As described previously [18], IHC staining was performed according to the manufacturer’s instructions. Tissue microarrays were incubated at 4 °C for overnight with anti-GRP78 polyclonal antibody (1:100, Santa Cruz, CA, USA). The immunoreactivity proportion was classified based on the percentage scores as < 5% (0), 5%–25% (1), 25–50% (2), 50–75% (3), and > 75% (4). The staining intensities based on intensity scores were classified as negative (0), weak (1), moderate (2), and strong (3). The final score was obtained by multiplying the percentage scores with the intensity scores. The total scores that ranged from 0 to 4 were defined as low group, and those that ranged from 5 to12 were defined as high group.

### Lentivirus-mediated siRNA construction and cell transfection

The lentivirus-mediated small interference RNA for GRP78 (siGRP78) and the negative control RNA (siNC) were designed and synthesized by Genepharma (Shanghai, China). The GRP78 siRNA sequence was: F: 5-GGUACUGCUUGAUGUAUGUTT-3, R: 5-ACAUACAUCAAGCAGUACCTT 3. The control siRNA sequence was: F: 5-UUCUCCGAACGUGUCACGUTT-3, R: 5-ACGUGACACGUUCGGAGAATT-3. For transfection experiments, the cells of ARO and FRO were plated in 6-well plates and siRNAs were transfected using Lipofectamine™ 2000 reagent (Invitrogen, USA) according to the manufacturer’s protocol for 24 h. The efficiency of gene silencing was further confirmed by western blotting in both the cell lines.

### Tunicamycin (TM) treatments

ARO and FRO cells at a density of 3 × 10^5^ cells/mL in a total volume of 3 mL were seeded in 6-well plates. After inoculation for 24 h, different concentrations of TM (0–1 μg/mL) were added and the cells were cultured for another 72 h. A parallelly untreated culture with same passage number as that of the adapted cultures was maintained as control culture, and the level of ER stress was further confirmed by western blotting in both the cells.

### Western blot assay

The cells were collected, the total protein was prepared in RIPA buffer (medium, Beyotime, China), and the cytoplasmic proteins were obtained using the Nuclear and Cytoplasmic Protein Extraction Kit (P0028, Beyotime, China). Western blotting was performed as described previously [20]. The antibodies used for western blotting were PERK, XBP1s and CLO1A1 (Abcam, USA), GRP78 and MMP13 (Santa Cruz, CA, USA), and β-actin (Beyotime Institute of Biotechnology Jiangsu, China).

### Cell proliferation

Cell counting kit-8 (CCK-8, Dojindo laboratories, Kumamoto, Japan) was used to measure cell proliferation. Cells were inoculated in 96-well plates, pre-incubated for 24 h and then for 0 h, 24 h, 48 h, 72 h or 96 h (5% CO_2_, 37 °C). At each time point, 10 μL CCK-8 solution was added to each well and the cells were further incubated for one to three hours. The absorbance (OD value) was measured using a microplate reader at 450 nm.

### Transwell migration assays

Transwell chambers were used for conducting migration assays in vitro as described previously [12]. The cells at a density of 5 × 10^4^ cells per well were seeded in the upper chamber (8 μm pore size, Corning, USA). After incubation for 24 h at 37 °C, the wells were washed with PBS thrice, fixed with methanol for 1 h and then stained with 1% crystal violet for 30 min. The cells on the upper surface of the filter were scraped off, and the cells on the lower surface of the filters were counted under a light microscope (Olympus BX51, Olympus) at 200× magnification in ten randomly selected fields. The experiment was repeated independently three times.

### GO Enrichment, KEGG pathway analysis of RNA-seq data

RNA‐sequencing data of GRP78 and si-GRP78 in ARO and FRO cells were tested by BGI (Shenzhen, China) [36]. The functional roles of differentially expressed genes (DEGs) were determined by the transcriptional profiles that are acquired using RNA-seq and GO and KEGG enrichment analyses. The GO database was used to analyze the functional enrichment of DEGs, and focused on the pathways enriched in the biological process, molecular function and cellular component that showed association with these genes. The KEGG pathway database was used to determine the enrichment of DEGs.

### GEPIA database for differential expression analysis ofMMP13 and CLO1A1 genes

Differential expression of MMP13 and CLO1A1 genes between 512 TC tissues and 337 normal tissues was acquired from the GEPIA (http://gepia.cancer-pku.cn/ ) [35]. In addition, the differential expression of MMP13 and CLO1A1 genes in terms of pathological stage (I–IV) was analyzed among the TC patients.

### Statistical analysis

Statistical significance was calculated with GraphPad Prism Software using one-way ANOVA with Tukey’s post-test and Student’s t-test. Data are presented as means ± SD. The values with *p* < 0.05 were considered to be statistically significant.

## Data Availability

The datasets generated during and/or analyses during the current study are available in GEPIA database.

## Abbreviations

TC: Thyroid cancer
PTC: Papillary thyroid carcinoma
FTC: Follicular thyroid carcinoma
PDTC: Poorly differentiated thyroid cancer
ATC: Anaplastic thyroid cancer
GRP78: Glucose-regulated protein78
GO: Gene ontology
KEGG: Kyoto Encyclopedia of Genes and Genomes
LN: Lymph node
ER: Endoplasmic reticulum
RNA-seq: RNA sequencing analysis
IHC: Immunohistochemistry
TM: Tunicamycin
DEGs: Differentially expressed genes
UPR: Unfolded protein response
ECM: Extracellular matrix
MMP: Matrix metalloproteinases
GEPIA: Gene Expression Profiling Interactive Analysis
